# Development of a strategy of physician-patient relationship for improving care for patients with disorders of sex development: a qualitative study: Desenvolvimento de uma estratégia de relacionamento médico-paciente para a melhoria do atendimento aos pacientes com desordens do desenvolvimento do sexo: um estudo qualitativo

**DOI:** 10.1590/1516-3180.2015.01750309

**Published:** 2016-06-03

**Authors:** Mariana Telles-Silveira, Felicia Knobloch, Claudio Elias Kater

**Affiliations:** I MSc. Postgraduate Fellow and Associate Psychologist, Division of Endocrinology, Department of Medicine, Escola Paulista de Medicina, Universidade Federal de São Paulo (EPM-Unifesp), São Paulo, SP, Brazil.; II PhD. Assistant Professor of Psychology, School of Human and Health Sciences, Pontifícia Universidade Católica de São Paulo, São Paulo, SP, Brazil.; III MD, PhD. Associate Professor of Medicine and Head of the Division of Endocrinology, Department of Medicine, Escola Paulista de Medicina, Universidade Federal de São Paulo (EPM-Unifesp), São Paulo, SP, Brazil.

**Keywords:** Disorders of sex development, Adrenal hyperplasia, congenital, Education, medical, Physician-patient relation, Interdisciplinary communication, Transtornos do desenvolvimento sexual, Hiperplasia suprarrenal congênita, Educação médica, Relações médico-paciente, Comunicação interdisciplinar

## Abstract

**CONTEXT AND OBJECTIVE:**

Care for patients with disorders of sex development (DSD) should be provided in integrated-care centers by a multidisciplinary team. Implementation of this project within the teaching clinic routine presents several challenges: 1) difficulties in relationships between the medical team and patients and their families; 2) age, ethnic and cultural differences; 3) DSD-related prejudice; and 4) physicians’ anxiety. We report on a psychologist’s work strategy that focused on creating arrangements that could contribute towards development of the relationship between the medical team and patients and their families, as a way of preparing the clinical staff to manage treatment of adult DSD patients.

**DESIGN AND SETTING:**

Prospective qualitative study.

**METHODS:**

Between February 2010 and April 2015, we conducted a qualitative study in the Adrenal Outpatient Clinic of Escola Paulista de Medicina (São Paulo, Brazil), based on interviews, team discussions and group dynamics with resident physicians, postgraduate students and attending physicians.

**RESULTS:**

Implementation of the project allowed residents to build a story of differentiated care for their patients, thus facilitating dialog between them and making it possible to address taboo topics. Sequential care provided by the same resident led patients to feel that their doctor cared for them, with individuality, continuity and a sense of interest in their story.

**CONCLUSION:**

Presence of a psychologist in the outpatient routine enabled inclusion of subjective factors in the routine of medical consultations, thus broadening the notion of healthcare for patients with DSD, facilitating bonds and providing support for difficulties faced.

## RESUMO

**CONTEXTO E OBJETIVO:**

Pacientes com desordens do desenvolvimento do sexo (DDS) e seus familiares devem ser atendidos em centros de atenção integral, por equipe multidisciplinar. A efetivação desse projeto no cotidiano da clínica-escola apresenta vários desafios: 1) dificuldades nas relações entre equipe médica, paciente e família, 2) diferenças etárias, étnicas e culturais, 3) preconceitos relacionados às DDS, e 4) angústia dos médicos. Relatamos o desenvolvimento de uma estratégia de trabalho do psicólogo, que teve como foco a criação de dispositivos que contribuíssem para o aprimoramento da relação entre equipe médica, paciente e família, preparando o *staff* clínico para administrar o tratamento de pacientes adultos com DDS.

**DESENHO E LOCAL:**

Estudo qualitativo prospectivo.

**MÉTODOS:**

De fevereiro de 2010 até abril de 2015, realizamos uma pesquisa qualitativa no Ambulatório de Adrenal da Escola Paulista de Medicina (São Paulo, Brasil), baseada em entrevistas, discussões de equipe e dinâmica de grupo com médicos residentes, pós-graduandos e assistentes.

**RESULTADOS:**

A implementação do projeto possibilitou aos residentes construir uma história de atendimento diferenciado com seus pacientes, facilitando o diálogo entre eles e permitindo que temas-tabus fossem abordados. O fato de ter sido atendido seguidamente pelo mesmo residente possibilitou ao paciente a sensação de cuidado, individualidade, continuidade e a sensação de que havia interesse, por parte do médico, sobre a sua história.

**CONCLUSÃO:**

A presença do psicólogo no cotidiano do ambulatório permitiu que aspectos subjetivos fossem incluídos na rotina das consultas médicas, ampliando a noção de saúde e cuidado aos pacientes com DDS, facilitando o vínculo e dando suporte para as dificuldades encontradas.

## INTRODUCTION

The physician-patient relationship and its models still constitute an object of study in several fields of science. The way in which this relationship develops within the medical context defines settings and attitudes, thereby building narratives about patients and their disease, and influencing these subjects’ experience.

Disorders of sex development (DSDs) are congenital conditions in which the development of chromosomal, gonadal or anatomical sex is atypical.^1^ DSDs are rare diseases that demand handling of hormonal, esthetic/surgical and emotional issues, since they involve matters that are central to the individual’s life, such as sex and gender definitions, reconstruction of malformed genitals, gender identity and sexuality. Thus, care for patients with DSDs and their families should be provided in integrated-care centers by a multidisciplinary team, taking into consideration particular issues such as age, religion, culture, socioeconomic status and education, among other factors.^1^^,^^2^^,^^3^

The treatment protocol for patients with DSDs has changed over the years,^4^ thus influencing the way in which physicians are prepared in order to handle these cases. During the “Money era” (1955-1990), the model for the dynamics between the medical staff and patients and their families was essentially based on policies of discretion and secrecy. This approach aimed to avoid discussion of the patient’s condition between the patient and his or her family, and it was justified by the idea that if the prevailing discourse was free from doubt, this would ensure development of a solid and healthy gender identity.^4^^,^^5^^,^^6^ Accordingly, that model was based on a “functionalist perspective”, and it expected the physician to take an active and decisive attitude, while the patient would have passive and submissive behavior.^7^

Today, the foundations for the treatment protocol are those described in the Chicago meeting of 2005.^1^ Provision of data on the disease and the treatment has begun to play a central role in handling these cases, thus demanding a new attitude from the physician, in which both sides expect transparency in their exchanges concerning the disease, with sharing of responsibilities between physicians, the families and the patients themselves.^1^^,^^4^

However, fragmentation of services (in particular, the disconnection between pediatric and adult clinics), the visits to different specialists that patients with DSDs have to go through and the turnover of professionals who work in the clinics of university hospitals (interns, residents, trainees and postgraduate students) constitute everyday challenges in clinical practice.^8^

One additional challenge intrinsic to any university medical school’s practice concerns the training of young doctors. Residents are graduate medical students who decide to follow and master a medical specialty. The in-training residence program is a highly stressful period in which many professionals are likely to develop burnout or depressive syndromes, and to require informative measures and help in caring for their mental status.^9^ The particular features of the several medical disciplines involved in attending DSDs patients require a wealth of diverse information and expertise from these in-training residents, as previously mentioned.^4^

In this context, we sensed the need to design a team intervention proposal in our endocrinology outpatient clinic, which specializes in caring for adult patients with DSDs, in an attempt to solve some specific challenges and to improve physician-patient/family relationships.

## OBJECTIVE

To report on a psychologist’s work strategy that aimed to establish arrangements that could contribute towards development of an effective relationship between the physician team and patients and their families, as a way of preparing the clinical staff to handle treatment of adult DSDs patients. The specific aims were to:

1. facilitate communication between physicians and adult patients; and
2. prepare and qualify young physicians to address and handle matters relating to sexuality, gender identity and sexual orientation.

## METHODS

During the period from February 2010 to April 2015, we followed three groups of endocrinology residents as they rotated in the Adrenal Outpatient Clinic of the Division of Endocrinology and Metabolism, Escola Paulista de Medicina, Universidade Federal de São Paulo (AOC-EPM).

During that period, 44 physicians taking part in medical school training and assistance activities provided attendance at the AOC-EPM: 36 in-training residents (third and fourth years of the course; averaging 11 residents/year, who rotated during the period), five postgraduate students and four assistant professors (fixed clinical group). Over the same period, care was provided for 55 patients with DSDs (40 with congenital adrenal hyperplasia due to 21-hydroxylase deficiency, seven due to 17-hydroxylase deficiency, three with total peripheral androgen resistance and five with other DSDs), in quarterly or biannual appointments. This group of patients corresponded to nearly 5% of the total number of patients followed in the clinic, which operates weekly.

In the present study, we assessed questionings, anxieties and doubts among the group of in-training residents who worked at the clinic over that period. We carried out the survey by using suitable psychological techniques:

1. group dynamics (conducted at the beginning and at the end of the in-training program);
2. annotated observations of the daily clinical routine; and
3. several medical school activities.

The latter included class lectures and clinical case discussions focusing on the psychological aspects of experiencing the disease and on matters such as sexuality, identity building, gender roles and ethical issues, among others.

The researcher (MTS) recorded all the activities right after they took place; she analyzed the data gathered by creating categories based on analysis of the transcripts, as per the five steps proposed by Pope^10^ (familiarization, identification of a thematic framework, indexing, charting and mapping/interpretation). She also analyzed the effects of patients’ discourse on the doctors, and vice-versa, taking psychoanalytical clinical listening as the reference.

Therefore, this report consisted of qualitative research that made use of observations, discussions and institutional interventions. The methodological guideline was research-action/intervention. This methodology was chosen through analysis on the subjective and emotional consequences for those involved in the clinic and interacting with the patients. For this reason, the researcher kept herself emotionally involved with what she intended to study and, in this way, the research field was composed of and through her presence.^10^^,^^11^^,^^12^^,^^13^^,^^14^^,^^15^^,^^16^

All the physicians and patients who actively took part in the activities signed a free and informed consent statement allowing the data gathered to be used in this study. The entire research project had gained consent from and was guaranteed by the head of the clinic, who monitored all the phases of development of the work. The project was approved by the Ethics Committee for Human Research of Universidade Federal de São Paulo, São Paulo, SP, Brazil (protocol #1842/10).

### Context

From the outset, we noticed that both the requests for psychological care and the participation in the discussion of a clinical case always brought with it some comments relating to aspects of the relationship that was being built between physician and patient. The following are examples:

*• “Do you think that girl wants to be a man or a woman?”*
*• “I don’t know how to approach the subject of corrective surgery, nor of relationships… at the same time, the patient tells me she is married. So I guess she does have a sex life…”*
*• “We believe the lack of continuity of the patient indicates that she wants her virilization; do you think that’s possible?*
*• “Does this patient like men or women?”*

One of the main problems that we detected, and one that involved the physician-patient relationship, lay in the difficulty in establishing bonds of trust. Since the patient met a different resident physician at every appointment, the physician did not have the opportunity to work through the questions he had raised even if all the staff discussed his questionings thoroughly in the clinical meetings.

The staff turnover and the clinic’s routine led to difficulties in the establishment of bonds. The physician could not answer questions that the patient brought, which made it difficult for him/her to build a story. Given the lack of continuity of his/her contact with the patient, the physician was objective and did not interact with the patient’s life story; he/she either focused only on the immediate demand, or on the question relating to adherence to treatment. In that model, the only option that remained for the physician was to gather objective data from examinations and compare these with data in the medical records.

Based on these considerations, and as a strategy to create new conditions for establishment of new work bonds, we have devised a clinical approach termed “in-training reference-resident”.

This strategy consisted of defining one in-training endocrine resident as the sole reference for caring for a specific DSD patient during a period of at least two years. Other physicians and graduating students could only follow the case together with the “in-training reference-resident” or during a clinical case presentation.

The “in-training reference-resident” was also responsible for scheduling future appointments in accordance with his/her shifts, in order to ensure priority for the patient. In situations of absence, he/she was to request assistance from another colleague within the group, and assemble information on the session afterwards. We explained and shared this procedure with the patient. At the end of the two-year residence, the “in-training reference-resident” would transfer his/her specific cases to a new colleague.

## RESULTS

The discourse analysis clearly showed that some categories were repeated:

1. uncertainty as to the patient’s adherence (disbelief in the physician’s words);
2. uncertainty regarding sexual orientation;
3. adaptation to gender identity;
4. sexuality; and
5. difficulty in handling the patient’s and their own subjective issues.

We considered that these comments were expressions of countertransference movements^17^^,^^18^ on the part of the medical staff, and therefore constituted the background to the patient’s clinical care.

Use of the “in-training reference-resident” approach enabled each resident to build a story of individualized care with his/her patients with DSDs. The fact that we directed one resident to each patient made the clinic’s functioning easier in many ways, thus allowing new developments for many problems that until then were apparently unsolvable. Consequently, the physicians were better oriented, which resulted in improved provision of care. Over the course of the process, the psychologist noticed that the residents started to feel more comfortable at every new session, especially when they felt empathy towards the case, or when they understood why a particular patient made them anxious.

During the assessment of the project, we often heard from the residents how impressed they were at the improvement in issues of treatment adherence. Moreover, they could assess how their patients were previously considered to be “protocols”, in which the examinations provided measurement and assessment of good or bad adherence. The discussion groups, lectures and group dynamics were crucial for supporting the acquisition of theoretical and subjective terms so that they could withstand the anxiety and tension raised by the situations that the patients brought to the sessions.

From the group dynamics, the residents reported how comforting it was to acknowledge that the difficulties inherent to the DSD clinic were felt by everyone. The exchange of experiences among them, in the various groups, favored building of a network of internal support, as well as maintenance of a common project, as can be inferred from their words:

*• “Wow, it is such a relief to hear what they’re saying, because I think I’ll have all that support; I think it will be a great learning experience for me. I came with lots of expectations and, by the look of it, there are many nuances; I think what I take from this session today is the importance of listening!”* (R3)
*• “ I don’t know how to handle a case like this; as a consequence, I don’t have doubts or questions; after all, I did not get in contact with that kind of patient; I don’t even know where to begin. Listening to the other residents makes me think I will learn a lot, and that the questions and answers will be built in the group!”* (R3)

During implementation of the “in-training reference-resident” project, the residents reported that they could relate the DSD’s theoretical-clinical dimension to the singularity of each patient’s story. Here are some samples from their words:

*• “I consider the Adrenal Outpatient Clinic one of the most difficult to work at and learn from. Every day, before seeing the patients, I try to prepare and study the pathologies, but when I am studying my patient X, it seems easier to understand the complex mechanisms of the disease”* (R4)
• “*When you see the patients in the project, you think about that particular patient; sometimes we want to get the parameters right and that is not always what the patient want. If I know him, I’ll have to consider other aspects, otherwise he will stop taking the medication, and I will blame it on him for being non-adherent!”* (R4)
*• “Our age doesn’t matter; there is always some tension in an appointment like that. We’re not used to deal with so many different variables. Each patient and each family are unique and they are always the ones who will teach us; it is that closer contact with the patient that will guarantee that we make a difference! Pathology is something we can learn, but relationship is something we have to build”.* (Attending physician).

The transition from one resident to another was an important observation to be improved. We noticed that it would be crucial to emphasize this transition, since it could potentially interfere with the progress of the treatments. Some patients established bonds with their doctor (through the mechanism of transference) in such a way that they did not want to leave that relationship, thus missing appointments with the new resident. By observing these behaviors, we were able to draw up measures involving the next resident’s turn, thus anticipating problems and sharing the process with the patients. The outcome from this “in-training reference-resident” strategy demonstrated that the organizational work and process influenced the physician-patient relationship and, therefore, the implications of treatment.

From the patients’ point of view, we could observe that the project also brought a series of counterparts, such as feelings of care, individuality and continuity, and especially a feeling that the physician was interested in them and their stories. During the follow-up, we no longer heard the patients complain that they did not understand the medical language. Many of them stated that they could not miss their appointments because they did not want to “drop the ball on their doctor”.

As a result, we noticed that physicians showing greater commitment had patients who were more engaged in their own treatment. Lastly, over these five years, we achieved effective improvements in medical care and in adherence to treatment.

## DISCUSSION

Through organizing the course of treatment of patients with DSDs better over the residence years, these young physicians could testify to concomitant development of a strong therapeutic alliance that was capable of changing behavioral patterns, which had not been considered possible until then. For example, patients had been missing their appointments, not taking their medications and not broaching topics that they initially judged themselves unable to address, etc. The closer contact enabled new ways of assessing patients’ suffering, while leaving some prejudice behind.

Implementation of the “in-training reference-resident” project had the aim of reducing fragmentation of the care provided for patients, as well as fostering conditions for the physician to establish a more human and singular form of interaction with the patients. Moreover, according to Campos,^19^ the medical project of a reference team has the purpose of creating and stimulating progressive production of a new standard of responsibility between the two players, thus leading to more responsibility and co-participation.

The residents were able to introduce and discuss taboo topics with the patients, which were not mentioned in the preceding sessions. From one session to the next, the residents had enough time not only to discuss the case with their superiors but also with the psychologist, so as to design a singular project with each patient and decrease the number of requests for psychological intervention, thereby favoring dialog. In other words, the topics that were addressed only by the psychologist could be worked through in the case discussions and could be dealt with directly by the “in-training reference-resident”, thus creating a less fragmented relationship and favoring a bond of trust.

Caprara and Franco^7^ and Adam and Herzlich,^20^ based on the works of Balint,^18^ Gadamer^21^ and others, stressed the importance of the bond established between physician and patient as a fundamental factor in ensuring greater effectiveness of the therapy established. Caprara’s analysis^7^ concerns the importance given to the doctor’s listening and attention given to the patient as something that can be compared to the healing effect of a medicine (“medicine-doctor”, in Balint’s expression).^18^

Several reference centers include the subject of Narrative Medicine in their medical programs or courses. This has the guiding principle of teaching physicians to listen and understand the stories of their patients. Narrative medicine has the aim of helping young physician to design listening tools that tell them what to do with patients’ stories. According to its founder, Charon,^22^ “if physicians do not know how to absorb and act on the stories they listen to, they will miss the opportunity to experience a real and therapeutically meaningful bond with their patients, who will thus be left adrift”^.22^

Therefore, creation of work tools that enable broadening of themes and favor development of relationships produces a direct improvement in patient care and decreases the rates of physician burnout, since there will be other people to talk to and share anxieties with.^23^

It is important to take into consideration the fact that, when adult patients start going to the clinic by themselves, without their parents or tutors, they feel pressured by the new questions that the doctor now asks them directly. Without intermediation, and often without previous preparation, we noticed that the diagnosis of anxiety returned, such that it was experienced a second time. In other words, the memories that remained in the patients’ bodies added to their present anxiety. When a physician deals with adult patients, this ends up triggering an unconscious association network, which surprises and, at the same time, scares patients. For this reason, treatment measures should cover those issues.

The “in-training reference-resident” project resulted in reorganization of the service, thus making it possible, both for physicians and for patients, to devise a new way of facing issues inherent to this clinic. We can state that there has been a change in quality of the physician-patient relationship, which has made the treatment process easier.

Furthermore, in one of the educational activities, we invited one of the residents who had formed part of the first group to give a class about the case he had worked on and followed up, reporting on the medical record, the challenges and his experience. That activity placed the new resident, who is now a specialist in endocrinology, in a different position, thus serving as a model for those who were arriving.

Finally, our study had one shortcoming: since our outpatient clinic treats patients with adrenal diseases in general and not exclusively DSDs, we were not able to conduct moments of reflection with the staff more often, because that would have taken up the time directed towards other activities in the clinic. In that sense, we believe that there is a need for a new routine in the clinic, centered on the DSD cases, so as to intensify the discussions, explore them in depth and better assess the residents’ learning process.

## CONCLUSION

The strategy described seemed to be useful for improving care for patients with DSDs, in spite of not being a controlled randomized clinical study. Both among the patients and the physicians, the proposal was highly valued and seems to have had a direct effect with regard to challenging issues, through favoring communication, enhancing bonds and giving greater esteem to these subjects’ lives.

## References

[1] Lee PA, Houk CP, Ahmed SF (2006). Consensus statement on management of intersex disorders. International Consensus Conference on Intersex. Pediatrics.

[2] Brauer S On the management of differences of sex development. Ethical issues relating to “intersexuality”.

[3] Hiort O, Birnbaum W, Marshall L (2014). Management of disorders of sex development. Nat Rev Endocrinol.

[4] Telles-Silveira M, Knobloch F, Kater CE (2015). Management framework paradigms for disorders of sex development. Arch Endocrinol Metab.

[5] Spinola-Castro AM (2005). A importância dos aspectos éticos e psicológicos na abordagem do intersexo [Importance of the ethical and psychological features in the intersex management]. Arq Bras Endocrinol Metab.

[6] Spinola-Castro AM, Maciel-Guerra AT, Guerra G (2010). Aspectos históricos e éticos dos distúrbios da diferenciação do sexo. Menino ou menina? Os distúrbios da diferenciação do sexo.

[7] Caprara A, Franco ALS (1999). A relação paciente-médico: para uma humanização da prática médica [The patient-physician relationship: towards humanization of medical practice]. Cad Saúde Pública.

[8] Kogan BA, Gardner M, Alpern AN (2012). Challenges of disorders of sex development: diverse perceptions across stakeholders. Horm Res Paediatr.

[9] Fagnani R, Obara CS, Macedo PC, Cítero VA, Nogueira-Martins LA (2004). Clinical and demographic profile of users of a mental health system for medical residents and other health professionals undergoing training at the Universidade Federal de São Paulo. Sao Paulo Med J.

[10] Pope C, Ziebland S, Mays N (2000). Qualitative research in health care. Analyzing qualitative data. BMJ.

[11] Barbier R (2002). A pesquisa-ação.

[12] Thiollent M (1987). Metodologia da pesquisa-ação.

[13] Patton MQ (1990). Qualitative evaluation and research methods.

[14] Milles MB, Huberman AM (1994). Qualitative data analysis.

[15] Mays N, Pope C (1995). Rigour and qualitative research. BMJ.

[16] Pope C, Mays N (1995). Reaching the parts other methods cannot reach: an introduction to qualitative methods in health and health services research. BMJ.

[17] Freud S (1969). As perspectivas futuras da terapia psicanalítica. Edição standard brasileira das obras psicológicas completas de Sigmund Freud.

[18] Balint M (1975). O médico, seu paciente e a doença.

[19] Campos GWS (1999). Equipes de referência e apoio especializado matricial: um ensaio sobre a reorganização do trabalho em saúde [Local reference teams and specialized matrix support: an essay about reorganizing work in health services]. Ciênc Saúde Coletiva.

[20] Adam P, Herzlich C (2001). Sociologia da doença e da medicina.

[21] Gadamer HG (2011). O caráter oculto da saúde.

[22] Charon R (2012). At the membranes of care: stories in narrative medicine. Acad Med.

[23] Charon R (2013). Narrative medicine in the international education of physicians. Presse Med.

